# Transcranial alternating current stimulation for schizophrenia: a systematic review of randomized controlled studies

**DOI:** 10.3389/fpsyt.2023.1308437

**Published:** 2024-01-11

**Authors:** Xin Wei, Zhan-Ming Shi, Xian-Jun Lan, Zhen-Juan Qin, Yu Mo, Hua-Wang Wu, Xing-Bing Huang, Qing-Bin Zeng, Li-Xia Luo, Xin-Hu Yang, Wei Zheng

**Affiliations:** ^1^The Brain Hospital of Guangxi Zhuang Autonomous Region, LiuZhou, China; ^2^Chongqing Jiangbei Mental Health Center, Chongqing, China; ^3^The Affiliated Brain Hospital of Guangzhou Medical University, Guangzhou, China; ^4^The Third People's Hospital of Foshan, Foshan, Guangdong, China; ^5^Chongqing Mental Health Center, Chongqing, China

**Keywords:** transcranial alternating current stimulation, schizophrenia, systematic review, negative symptoms, randomized clinical trial

## Abstract

**Background:**

In randomized clinical trials (RCTs) investigating the application of transcranial alternating current stimulation (tACS) in schizophrenia, inconsistent results have been reported. The purpose of this exploratory systematic review of RCTs was to evaluate tACS as an adjunct treatment for patients with schizophrenia based on its therapeutic effects, tolerability, and safety.

**Methods:**

Our analysis included RCTs that evaluated adjunctive tACS’ effectiveness, tolerability, and safety in schizophrenia patients. Three independent authors extracted data and synthesized it using RevMan 5.3 software.

**Results:**

Three RCTs involving 76 patients with schizophrenia were encompassed in the analysis, with 40 participants receiving active tACS and 36 receiving sham tACS. Our study revealed a significant superiority of active tACS over sham tACS in improving total psychopathology (standardized mean difference [SMD] = −0.61, 95% confidence interval [CI]: −1.12, −0.10; *I*^2^ = 16%, *p* = 0.02) and negative psychopathology (SMD = −0.65, 95% CI: −1.11, −0.18; *I*^2^ = 0%, *p* = 0.007) in schizophrenia. The two groups, however, showed no significant differences in positive psychopathology, general psychopathology, or auditory hallucinations (all *p* > 0.05). Two RCTs examined the neurocognitive effects of tACS, yielding varied findings. Both groups demonstrated similar rates of discontinuation due to any reason and adverse events (all *p* > 0.05).

**Conclusion:**

Adjunctive tACS is promising as a viable approach for mitigating total and negative psychopathology in individuals diagnosed with schizophrenia. However, to gain a more comprehensive understanding of tACS’s therapeutic effects in schizophrenia, it is imperative to conduct extensive, meticulously planned, and well-documented RCTs.

## Introduction

Schizophrenia is a debilitating condition characterized by impaired cognitive, emotional, and thinking functions. It is frequently a chronic and persistent illness (1, 2). Besides constituting a substantial disability, schizophrenia burdens families and society significantly while profoundly impacting the quality of life for affected individuals (3). The inadequacy of current treatments for schizophrenia could lead to the emergence of aggressive and violent behaviors among patients, exacerbating societal issues and intensifying the associated stigma (4–6). Enhancements in schizophrenia treatment could have broader implications for public health.

Currently, therapeutic strategies for schizophrenia encompass pharmacological, psychological, and physical interventions. However, pharmacological treatments encounter challenges, including inadequate efficacy in certain patients, resulting in treatment-resistant forms of schizophrenia (7). Cognitive-behavioral therapy (CBT), a prominent psychological intervention, has been extensively utilized for schizophrenia treatment (8). However, its implementation requires the patients to be stable, entails extended intervention durations, is accompanied by high costs, and demands substantial patient cooperation (9). There has been a growing utilization of non-pharmacological interventions to augment the effectiveness of antipsychotic treatments within clinical settings. These interventions encompass adjunctive non-invasive brain stimulation (NIBS) techniques, including electroconvulsive therapy (ECT) (10, 11), transcranial magnetic stimulation (TMS) (12), magnetic seizure therapy (MST) (13), transcranial direct current stimulation (tDCS), (14), and transcranial alternating current stimulation (tACS) (15).

Over the past two decades, tACS, an electrical brain stimulation method, has gained widespread acceptance within the medical field (16). Its applications include the treatment of mental disorders (17), attention enhancement (18), cognitive ability improvement (19), and sleep pattern regulation (20). Following extensive research and development, tACS technology will become a vital tool for tailoring medical treatments in these areas (21). Contrary to traditional techniques such as tDCS and TMS, tACS completely avoids sensory stimulation, it employs sinusoidal and biphasic alternating currents to stimulate cortical neurons, thereby regulating intrinsic brain oscillations and governing the synchronization and desynchronization of neural activity within the cerebral cortex. Consequently, this modulation of cortical excitability and brain function occurs (22). tACS potentially induces synaptic plasticity changes and regulates neurotransmitter levels (23, 24), enhancing long-term cognitive function and alleviating psychiatric symptoms. Consequently, this technology demonstrates significant potential for further development within schizophrenia.

The results of a randomized single-blind study suggest that gamma-tACS could be more effective than tDCS in improving working memory in people with schizophrenia (25). While in three recent double-blinded randomized controlled trials (RCTs), tACS has been examined for its feasibility, efficacy, and safety in the treatment of adult schizophrenia patients (26–28), their results have been varied. Chang et al. observed more substantial decreases in Positive and Negative Syndrome Scale (PANSS) negative subscale scores following theta-frequency tACS (θ-tACS) stimulation in the active condition (13.84%) compared to the sham condition (3.78%), accompanied by significant effect size (26). However, the two remaining RCTs examining alpha-frequency tACS (α-tACS) in patients with schizophrenia did not find significant improvements in total psychopathology (27, 28).

A systematic review aimed to detect the impact of tACS on cognition, depression, and schizophrenia (29), however, it included only limited studies (2 RCTs, *n* = 51) examining the impact of tACS on schizophrenia, resulting in insufficient statistical power. The objective of this exploratory systematic review, incorporating a recent RCT (28), was to acquire more substantial evidence about the effectiveness and safety of adjunctive tACS when combined with antipsychotic medications.

## Materials and methods

### Search strategy and selection criteria

Three investigators (ZMS, ZJQ, and XJL) independently searched four international databases (PubMed, EMBASE, PsycINFO, and Cochrane Library) from the database’s inception to February 6, 2023. The search terms used were: (“transcranial alternating current stimulation” OR tACS) AND (schizophrenia [MeSH] OR schizophrenic disorder OR disorder, schizophrenic OR schizophrenic disorders OR schizophrenia OR dementia praecox). Furthermore, the researchers manually examined the reference lists of the included studies (26–28), systematic review (29) and scoping review (30) on tACS for patients with schizophrenia to identify any missing RCTs.

Following the Preferred Reporting Items for Systematic Reviews and Meta-Analyses (PRISMA) guidelines, studies meeting the following PICOS criteria were included (31). ***P***articipants: Individuals with schizophrenia or schizoaffective disorder, regardless of diagnostic criteria. ***I***ntervention versus ***C***omparison: Antipsychotic medications combined with active tACS versus those combined with sham tACS. ***O***utcomes: The primary outcome assessed was the post-tACS change in total psychopathology, measured using standardized instruments such as the PANSS (32) or the Brief Psychiatric Rating Scale (BPRS) (33). Secondary outcomes included positive, negative, and general psychopathology scores on the PANSS or BPRS, the Scale for the Assessment of Negative Symptoms (SANS) or the Scale for the Assessment of Positive Symptoms (SAPS), auditory hallucinations scores measured using the Auditory Hallucination Rating Scale (AHRS) (34), cognitive function, discontinuation for any reason, and adverse events. When a study employed multiple measures to assess positive and negative psychopathology, preference was given to PANSS subscale scores to minimize heterogeneity. ***S***tudy: Only published double-blinded RCTs on adjunctive tACS for patients with schizophrenia were eligible for inclusion. A randomized single-blind study focusing on γ-tACS for schizophrenia was notably excluded (25). We also excluded studies comparing active tACS with tDCS or other forms of physical therapy, review articles, and case reports/series.

### Data extraction

Data were extracted from each included RCT by three independent researchers (ZJQ, XJL, and ZMS). Any discrepancies were resolved through collaborative discussions involving a senior author (WZ). A standardized form was used to collect information, including authorship details, publication year, study design, tACS protocol, and primary and secondary outcomes. The original study authors were contacted to obtain missing data when further information was required. Only pre-crossover data were extracted if the eligible RCT had a crossover design (28).

### Study quality assessment

To evaluate the quality of the RCTs, three independent researchers (ZJQ, XJL, and ZMS) used the Jadad scale (35) and the Cochrane risk of bias tool (36). RCTs with a Jadad score of ≥3 were categorized as “high quality” (37). For all meta-analyzable outcomes, the Grading of Recommendations, Assessment, Development, and Evaluation (GRADE) system was employed.

### Statistical analyses

We conducted statistical analyses with RevMan software (version 5.3) using random effects models (38). Risk ratio (RR) and standard mean difference (SMD) were computed for dichotomous and continuous outcomes, respectively, along with 95% confidence intervals (CIs). Heterogeneity among the studies was assessed using Cochrane’s *Q* and *I*^2^ test, with *Q* < 0.1 or *I*^2^ ≥ 50% indicating significant heterogeneity (39). For primary outcomes with *I*^2^ ≥ 50%, sensitivity and subgroup analyses were conducted to elucidate the heterogeneity. In all analyses, publication bias was assessed with funnel plots and Egger’s test (40), with 5% significance at two-tailed *p*-values.

## Results

### Literature search

**Figure 1:**
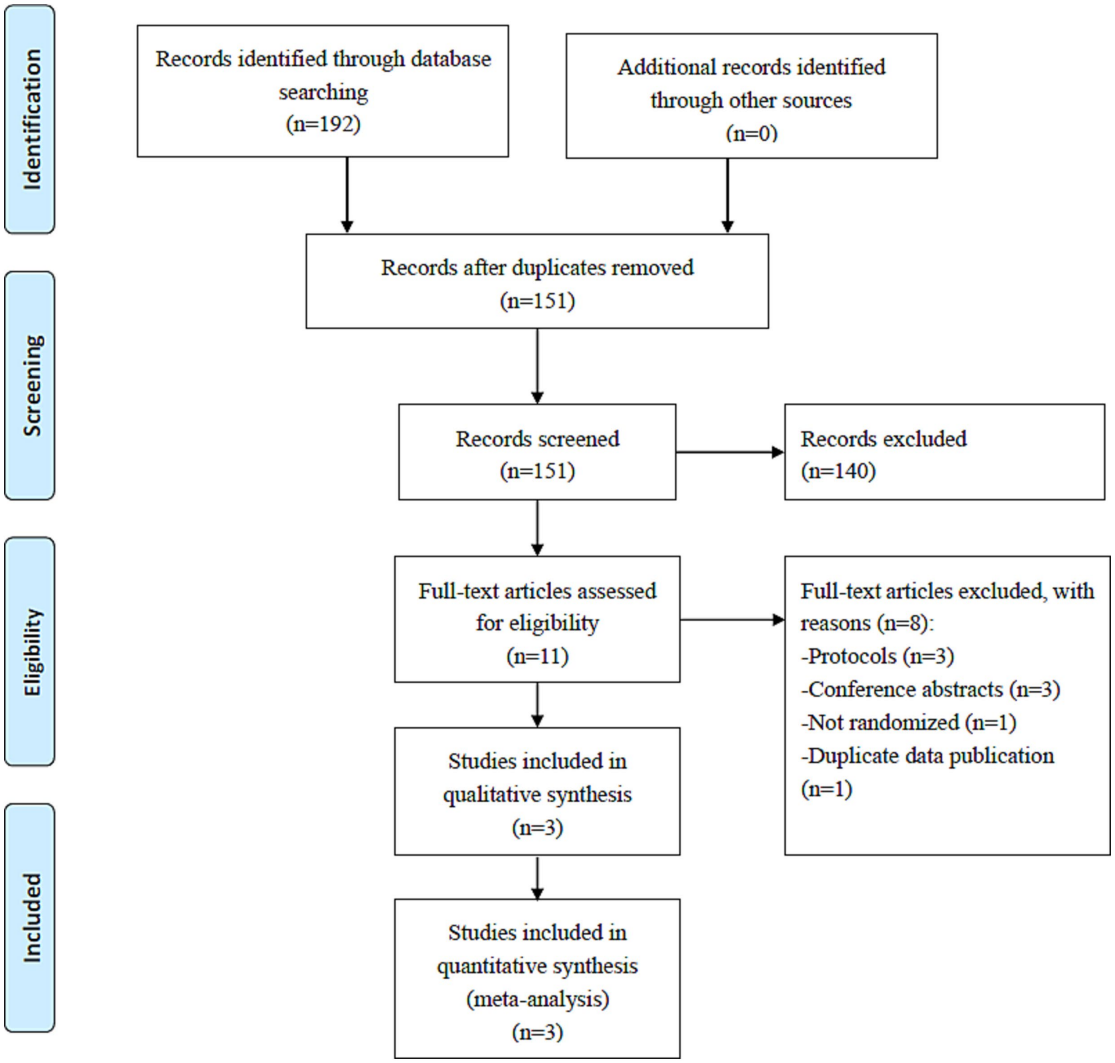
PRISMA flow diagram.

Following the search strategy, 192 trials were retrieved. On screening the titles, abstracts, and full texts, three RCTs that met the inclusion criteria (26–28) were analyzed in this meta-analysis (Figure 1).

### Study characteristics

Table 1 summarizes the participant characteristics and tACS parameters of the three included RCTs. These RCTs (*n* = 76) that were published between 2018 and 2022 compared active tACS (*n* = 40) and sham tACS (*n* = 36) in patients with schizophrenia or schizoaffective disorder. Among them, two RCTs (66.6%) were conducted in the United States, and one (33.3%) was performed in China (Table 1). In this study, the average age of participants was 41.5 years old (range 18–70) and 59.2% of participants were men (range 50–73%). All three RCTs employed a 2 mA current intensity (6 Hz-tACS in one RCT or 10 Hz-tACS in two RCTs), with stimulation sessions scheduled either twice daily for 20 min (26, 27) or once daily for 40 min over five consecutive days (28). The follow-up period for the included three RCTs ranged from one (2 RCTs) (26, 27) to two months (1 RCT) (28).

**Table 1:** Participant characteristics and tACS parameters for each included study.

Studies (country)	N^a^	-Blinding -Analyses	-Diagnosis -Diagnostic Criteria -Setting	Illness duration (yrs)^b^	Age range^b^: (yrs)	Gender^b^: male (%)	Montage anode (active/sham) -Stimulation electrodes -Return electrode	tACS stimulation frequency, intensity, and APs dosages (mg/day); Number of patients (n)	Follow-up period (months)	Treatment duration (days)^c^	-Length (min) -Number of sessions (n/day)	Jadad score
(26) (China)	36	-DB -ITT	-Schizophrenia or schizoaffective disorder -DSM-5 -NR	16.3	42.5 (20–65)	18 (50.0)	-F1, F5, AF3, FC3, P1, P5, CP3, PO3 -CPz, FCz^d^	1. Active tACS (6 Hz, 2 mA) + APs (NR^e^); *n* = 18 2. Sham tACS (a tiny current pulse that 110 μA over 15 ms) + APs (NR^e^); *n* = 18	1	5	-20 −10 (2/day)	5
(27) (United States)	15	-DB -OC	-Schizophrenia or schizoaffective disorder -DSM-IV -Outpatients	NR	43.2 (NR)	11 (73.3)	-F3, Fp1, T3, P3 -Cz^f^	Active tACS (10 Hz, 2 mA) + APs (NR^g^); *n* = 8 Sham tACS (10 s of ramp-in to 20 s of 10 Hz tACS, with a ramp-out of 10 s for a total of 40 s of stimulation) + APs (NR^g^); *n* = 7	1	5	−20 −10 (2/day)	3
(28) (United States)	25	-DB -ITT	-Schizophrenia or schizoaffective disorder -DSM-IV -Outpatients	NR	39.0 (18–70)	16 (64.0)	-F3, Fp1, T3, P3 -Cz^f^	Active tACS (10 Hz, 2 mA) + APs (NR^g^); *n* = 14 Sham tACS (10 s of ramp-in to 20 s of 10 Hz tACS, with a ramp-out of 10 s for a total of 40 s of stimulation) + APs (NR^g^); *n* = 11	2	5	−40 −5 (1/day)	5

a Data were extracted based on random assignment and compliance with the selection criteria.

b Available data were extracted by considering the mean baseline value from each included trial.

c The treatment period was defined as spanning from initiating the first tACS treatment to the endpoint of the final tACS session.

d The placement of the return electrode was determined using the international 10–10 placement system.

e An adequate therapeutic dose of antipsychotics was administered as needed; several participants received two antipsychotic medications.

f The location of the return electrode was determined using the international 10–20 placement system.

g At least two different antipsychotic agents of adequate doses were used as needed.

APs, antipsychotics; DB, double blind; DSM-IV, Diagnostic and Statistical Manual of Mental Disorders 4th edition; DSM-V, Diagnostic and Statistical Manual of Mental Disorders 5th edition; ITT, intent-to-treat; L-DLPFC, left dorsolateral prefrontal cortex; L-TPJ, left temporoparietal junction; min = minutes; NR, not reported; OC, observed cases; tACS, transcranial alternating current stimulation; yrs, years.

### Quality assessment

**Figure 2:**
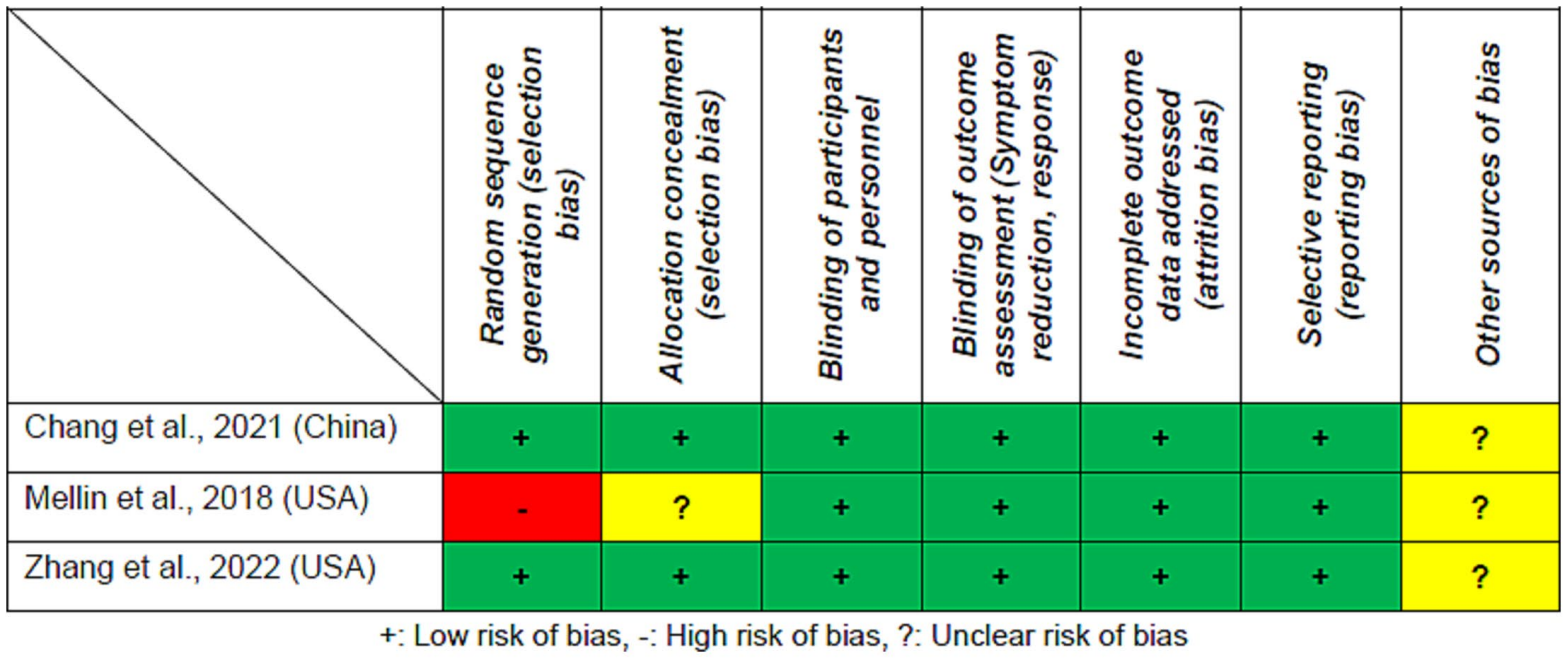
Cochrane risk of bias.

Regarding random sequence generation and allocation concealment, two RCTs (66.7%) were rated as ‘low risk’ (Figure 2). The blinding of participants and personnel, blinding of outcome assessment, incomplete outcome data, and selective reporting of RCTs were all rated as being ‘low risk’. The mean Jadad score was 4.3 (range = 3–5), classifying all included RCTs as high-quality studies (Jadad score ≥ 3) (Table 1). According to the GRADE approach (Supplementary Table 1), evidence quality was moderate for all primary and secondary outcomes (100%).

### Psychotic psychopathology

**Figure 3:**
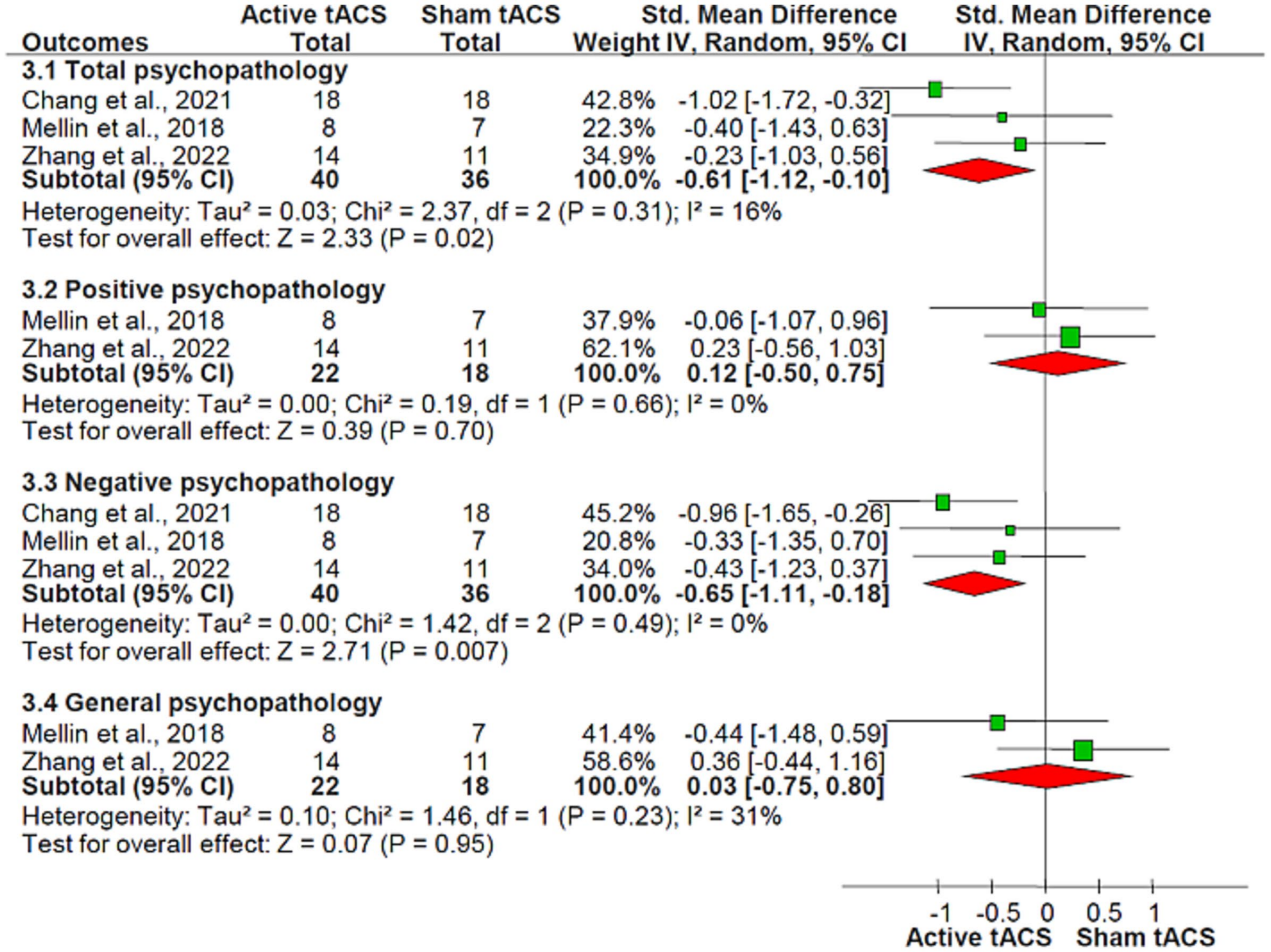
tACS for schizophrenia: the forest plot of overall, positive, negative, and general psychopathology scores measured by PANSS Abbreviations: CI, confidence interval; PANSS, positive and negative syndrome scale; tACS, transcranial alternating current stimulation.

Adjunctive active tACS demonstrated superiority over the sham tACS group in improving total psychopathology (SMD = −0.61, 95% CI: −1.12, −0.10; *I*^2^ = 16%, *p* = 0.02) as measured by the PANSS, and in decreasing negative psychopathology (SMD = −0.65, 95% CI: −1.11, −0.18; *I*^2^ = 0%, *p* = 0.007), calculated using the PANSS-negative symptoms subscale. However, no significant differences were observed between groups in terms of changes in positive psychopathology (2 RCTs, *n* = 40, SMD = 0.12, 95% CI: −0.50, 0.75; *I*^2^ = 0%, *p* = 0.70, Figure 3) (27, 28) assessed by the PANSS-positive symptoms subscale, general psychopathology (2 RCTs, *n* = 40, SMD = 0.03, 95% CI: −0.75, 0.80; *I*^2^ = 31%, *p* = 0.95, Figure 3) (27, 28), measured with the PANSS-general psychopathology subscale, and auditory hallucinations (2 RCTs, *n* = 40, SMD = −0.04, 95% CI: −0.66, 0.58; *I*^2^ = 0%, *p* = 0.90; Table 2) (27, 28) as measured by the AHRS.

**Table 2:** tACS for schizophrenia: secondary outcomes.

Variables	Study (subjects)	SMDs/RRs (95% CI)	*I*^2^ (%)	*p*
*Auditory hallucination symptom*				
Auditory hallucination symptom measured by the AHRS	2 (40)	−0.04 (−0.66, 0.58)	0	0.90
*Discontinuation rate*				
Discontinuation due to any reason	3 (76)	2.67 (0.13, 56.63)	NA	0.53

AHRS, Auditory Hallucination Rating Scale; CI, confidence interval; NA, not applicable; RRs, risk ratios; SMDs, standardized mean differences; tACS, transcranial alternating current stimulation.

### Neurocognitive function

Two out of three RCTs (66.7%) investigated the impact of adjunctive tACS on cognitive function in schizophrenia patients, yielding mixed results (26, 27) (Supplemental Table 2). According to Chang et al.’s study (26), active tACS improves working memory significantly more than sham tACS when measured with dual n-back tasks, while Mellin et al.’s study (27) did not report such an improvement.

### Discontinuation rate and adverse events

There were no significant differences between the groups in terms of discontinuation due to any reason (RR = 2.67, 95% CI: 0.13, 56.63; *I*^2^ = not applicable, *p* = 0.53; Table 2). As indicated in Supplementary Table 3, adverse events were assessed using an adverse-effects questionnaire, and the most commonly reported ones associated with tACS in the included RCTs encompassed tingling, drowsiness, scalp pain, dizziness, difficulty concentrating, itching, headaches, and a burning sensation. No relevant significant differences were detected (all *p* > 0.05).

### Publication bias

As fewer than 10 RCTs were included, publication bias could not be analyzed as recommended (41).

## Discussion

This exploratory systematic review encompassed three double-blinded RCTs, encompassing 76 individuals diagnosed with schizophrenia. The primary findings demonstrated the superiority of active tACS over sham tACS in effectively addressing the total and negative psychopathological symptoms. Negative symptoms are inherent to schizophrenia and correlated with neurocognitive impairments (42). tACS significantly improved negative symptoms of schizophrenia, which is consistent with several other NIBS approaches, including tDCS (43) and repetitive TMS (rTMS) (44). However, it is crucial to acknowledge that the evidence quality for total and negative psychopathology presented in this exploratory systematic review is low, likely attributable to the limited sample size, which ranged from 15 to 36 participants. tACS appeared ineffective in treating positive psychopathology, general psychopathology, and auditory hallucinations inherent to schizophrenia. Only two RCTs (66.7%) investigated the cognitive effects of tACS in patients with schizophrenia, yielding inconsistent results (26, 27). This exploratory systematic review suggests that tACS is safe and well-tolerated for treating schizophrenia.

The incorporated RCTs with sample sizes ranging from 15 to 36 were published within the last five years, signifying the novelty and clinical significance of tACS in schizophrenia. The findings from this meta-analysis suggest that tACS could be a viable non-pharmacological intervention for individuals with schizophrenia. Beyond its effectiveness in improving total and negative psychopathology of schizophrenia, tACS has been explored as a treatment for major depression (MDD) (45), chronic insomnia (46), attention deficit hyperactivity disorder (ADHD) (47), and pain disorders (48). For instance, a recent meta-analysis has determined that tACS effectively alleviates depression symptoms in individuals with MDD (45) while ensuring safety. Furthermore, a systematic review of RCTs (*n* = 73) has demonstrated tACS as an effective and safe therapeutic approach for chronic insomnia (46). However, this systematic review found that tACS does not demonstrate efficacy in treating auditory hallucinations in individuals with schizophrenia, as assessed by the AHRS. Moreover, a recent meta-analysis encompassing eight RCTs (*n* = 329) suggests that a regimen of twice-daily stimulation or ten sessions of tDCS is necessary to improve auditory hallucination symptoms, as assessed by the AHRS (49). However, a comparative study revealed that there was no significant disparity in terms of safety, tolerability, and efficacy for the treatment of schizophrenia between the tACS and tDCS groups (27).

The potential neuronal mechanism underlying the efficacy of tACS in ameliorating negative symptoms could be attributed to its ability to entrain brain network oscillations (50). These adverse symptoms are commonly linked with impairments in cognitive function and dysregulated dopaminergic transmission within the mesocorticolimbic pathway, including the ventral tegmental area (VTA), ventral striatum (*VS*), hippocampus (HP), and prefrontal cortex (PFC) (26). An aberrant functional coupling between the PFC, VTA, and HP is believed to play a crucial role in the manifestation of these abnormalities (51, 52). Accumulating research suggests that theta-rhythm oscillations coordinate neuronal activity within the PFC-VTA-HP axis when engaged in cognitive processes, such as working memory (53, 54). This phenomenon is illustrated by rTMS. Intermittent theta-burst stimulation (iTBS) targets the left dorsolateral prefrontal cortex (DLPFC) at a theta rhythm. iTBS has demonstrated the ability to alleviate negative symptoms while influencing neural transmission in the PFC, *VS*, and HP (26, 55, 56). Chang et al. found that θ-tACS could modulate frontoparietal networks (26) while treating schizophrenia. The potential neuronal mechanisms underlying the clinical effectiveness of tACS in individuals with schizophrenia could involve synchronizing intrinsic brain oscillations with the stimulation frequency and establishing long-range oscillatory connections between distant brain regions, such as the PFC-VTA-HP axis.

Another primary objective of alternative NIBS techniques, such as tDCS and TMS, is to assess their neurocognitive function. For instance, a previous meta-analysis revealed that the supplementary use of tDCS demonstrates a significant therapeutic impact on ameliorating working memory impairments in individuals with schizophrenia (14). There is a common link between major mental disorders and cognitive impairments, especially schizophrenia. However, only two RCTs (66.6%, 2/3), with inconsistent findings, have evaluated the neurocognitive function of tACS in schizophrenia. In this systematic review, the rates of discontinuation and adverse events were similar across the active and sham groups, indicating that tACS may be a safe and well-tolerated treatment strategy for patients with schizophrenia in clinical practice. The electrical stimulation techniques (tDCS and tACS) possess several advantageous features such as cost-effectiveness, portability, suitability for home use, and compatibility with training or rehabilitation interventions (57). Importantly, as compared with tDCS, tACS seemed to cause fewer adverse effects (58). Therefore, tACS has been also proven as a safe intervention for patients suffering from MDD (45), chronic insomnia (46), and healthy participants (59).

Several limitations are present in this exploratory systematic review. First, analyses were limited due to the small sample size (*n* = 76) and the number of RCTs included (3 RCTs), the current findings are still preliminary and exploratory. Second, unpublished RCTs with negative results can affect the interpretation of these findings since publication bias cannot be conducted in this exploratory systematic review. Third, the parameters of tACS differ across the included three RCTs in this exploratory systematic review. Consequently, there is a need for determining the optimal parameters (e.g., frequency of daily sessions and total sessions) of tACS for schizophrenia. Fourth, the included studies did not implement long-term follow-up (e.g., beyond 2 months) despite the fact that maintaining the antipsychotic effects remains a major concern for tACS. Finally, the protocol of this systematic review was not registered.

## Conclusion

The use of adjunctive tACS shows promise as a viable approach to alleviate overall and negative psychopathology in individuals diagnosed with schizophrenia. However, it is imperative to conduct extensive, meticulously planned, and well-documented RCTs to better understand tACS’s therapeutic effects in schizophrenia. Additional research is necessary to investigate the optimal parameters for tACS, encompassing the identification of the most effective frequency for daily sessions, the total number of sessions, and the temporal distribution of treatment days.

## Data availability statement

The original contributions presented in the study are included in the article/Supplementary material, further inquiries can be directed to the corresponding authors.

## Author contributions

XW: Methodology, Writing – original draft. Z-MS: Data curation, Writing – original draft. X-JL: Data curation, Writing – original draft. Z-JQ: Formal analysis, Writing – review & editing. YM: Data curation, Writing – original draft. H-WW: Investigation, Software, Writing – review & editing. X-BH: Conceptualization, Investigation, Writing – review & editing. Q-BZ: Conceptualization, Data curation, Writing – review & editing. L-XL: Data curation, Formal analysis, Writing – review & editing. X-HY: Methodology, Resources, Software, Supervision, Validation, Visualization, Writing – review & editing. WZ: Conceptualization, Investigation, Methodology, Project administration, Resources, Supervision, Validation, Visualization, Writing – review & editing.
