# Supplementary material for: Transcranial alternating current stimulation for schizophrenia: a systematic review of randomized controlled studies
Source: Front Psychiatry. 2024 Jan 11;14:1308437. doi: 10.3389/fpsyt.2023.1308437 (PMC10808327; doi:10.3389/fpsyt.2023.1308437)
Supplement: Supplementary file 1 [file Table_1.DOCX]

**Supplemental Table 1. GRADE analyses: adjunctive tACS for schizophrenia.**

| **Primary and** *secondary outcomes* | **N (subjects)** | **Risk of bias** | **Inconsistency** | **Indirectness** | **Imprecision** | **Publication bias** | **Large effect** | **Overall quality of evidence^a^** |
| --- | --- | --- | --- | --- | --- | --- | --- | --- |
| **Total psychopathology** | 3 (76) | No^b^ | No | No | Serious^c^ | Undetected | No | +/+/+/-; Moderate |
| *Positive psychopathology* | 2 (40) | No^b^ | No | No | Serious^c^ | Undetected | No | +/+/+/-; Moderate |
| *Negative psychopathology* | 3 (76) | No^b^ | No | No | Serious^c^ | Undetected | No | +/+/+/-; Moderate |
| *General psychopathology* | 2 (40) | No^b^ | No | No | Serious^c^ | Undetected | No | +/+/+/-; Moderate |
| *Auditory hallucination symptom* | 2 (40) | No^b^ | No | No | Serious^c^ | Undetected | No | +/+/+/-; Moderate |
| *Discontinuation due to any reason* | 3 (76) | No^b^ | No | No | Serious^c^ | Undetected | No | +/+/+/-; Moderate |
| ^a^ GRADE Working Group grades of evidence: High quality=further research is very unlikely to change our confidence in the estimate of effect. Moderate quality=further research is likely to have an important impact on our confidence in the estimate of effect and may change the estimate. Low quality=further research is very likely to have an important impact on our confidence in the estimate of effect and is likely to change the estimate. Very low quality=we are very uncertain about the estimate. ^b^ All included studies were double-blind RCTs which were rated as high-quality studies.  ^c^ For continuous outcomes, N<400. For dichotomous outcomes, N<300.  Abbreviations: GRADE=Grading of Recommendations Assessment, Development, and Evaluation; tACS=transcranial alternating current stimulation. | | | | | | | | |

**Supplemental Table 2. tACS for schizophrenia: neurocognitive function.**

| **Studies** | **Neurocognitive function** | **Findings** |
| --- | --- | --- |
| Chang et al., 2021 (China) | Dual n-back task  WCST  CPT-II  Digit span  FTT  TOL  CTT  Stroop interference test | Compared with sham stimulation, tACS could significantly improve neurocognitive function measured by the dual n-back task in patients with schizophrenia. No significant differences were identified between the two groups regarding neurocognitive function assessed by the WSCT, CPT-II, Digit span, FTT, TOL, CTT, and stroop interference test. |
| Mellin et al., 2018 (USA) | BACS | No significant differences were observed between groups in neurocognitive function measured by BACS. |
| Zhang et al., 2022 (USA) | NR | NR |
| Abbreviations: BACS=Brief Assessment of Cognition in Schizophrenia; CPT-II=Connors’ Continuous Performance Test-2nd Edition; CTT=Color Trails Test; FTT=Finger Tapping Test; NR=not reported; tACS=transcranial alternating current stimulation; TOL=Tower of London test; WCST=Wisconsin Card Sorting Test. | | |

**Supplemental Table 3. tACS for schizophrenia: adverse events.**

| **Studies** | **Adverse events** | **Findings** |
| --- | --- | --- |
| Chang et al., 2021 (China) | Adverse-effects questionnaire | No significant differences were identified between the active and sham tACS groups regarding the frequency of burning sensation, dizziness, headache, itching, scalp pain, sleepiness, tingling, and difficulty focusing. |
| Mellin et al., 2018 (USA) | Adverse-effects questionnaire | No significant differences were identified between the active and sham tACS groups regarding the average score of burning sensation, flickering lights, headache, improved mood, itching, scalp pain, sleepiness, scalp pain, and difficulty focusing. |
| Zhang et al., 2022 (USA) | Adverse-effects questionnaire | No significant differences were found between the active and sham tACS groups regarding the average score of burning sensation, dizziness, flickering lights, headache, improved mood, itching, local redness, neck pain, ring/buzzing noise, scalp pain, sleepiness, tingling, difficulty focusing, and worsening of mood. |
| Abbreviations: tACS=transcranial alternating current stimulation. | | |
